# Success with antiretroviral treatment for children in Kigali, Rwanda: Experience with health center/nurse-based care

**DOI:** 10.1186/1471-2431-8-39

**Published:** 2008-10-02

**Authors:** Johan van Griensven, Ludwig De Naeyer, Jeanine Uwera, Anita Asiimwe, Claire Gazille, Tony Reid

**Affiliations:** 1 Médecins Sans Frontières, Kigali, Rwanda; 2 Interact Rwanda, Kigali, Rwanda; 3 Treatment and Research AIDS Center (TRAC), Kigali, Rwanda; 4 Medical department, Operational Centre Brussels, Médecins Sans Frontières, Brussels, Belgium

## Abstract

**Background:**

Although a number of studies have shown good results in treating children with antiretroviral drugs (ARVs) in hospital settings, there is limited published information on results in pediatric programs that are nurse-centered and based in health centers, in particular on the psychosocial aspects of care.

**Methods:**

Program treatment and outcome data were reported from two government-run health centers that were supported by Médecins Sans Frontières (MSF) in Kigali, Rwanda between October 2003 and June 2007. Interviews were held with health center staff and MSF program records were reviewed to describe the organization of the program. Important aspects included adequate training and supervision of nurses to manage ARV treatment. The program also emphasized family-centered care addressing the psychosocial needs of both caregivers and children to encourage early diagnosis, good adherence and follow-up.

**Results:**

A total of 315 children (< 15 years) were started on ARVs, at a median age of 7.2 years (range: 0.7–14.9). Sixty percent were in WHO clinical stage I/II, with a median CD4% of 14%. Eighty-nine percent (n = 281) started a stavudine-containing regimen, mainly using the adult fixed-dose combination. The median follow-up time after ARV initiation was 2 years (interquartile range 1.2–2.6). Eighty-four percent (n = 265) of children were still on treatment in the program. Thirty (9.5%) were transferred out, eight (2.6%) died and 12 (3.8%) were lost to follow-up. An important feature of the study was that viral loads were done at a median time period of 18 months after starting ARVs and were available for 87% of the children. Of the 174 samples, VL was < 400 copies/ml in 82.8% (n = 144). Two children were started on second-line ARVs. Treatment was changed due to toxicity for 26 children (8.3%), mainly related to nevirapine.

**Conclusion:**

This report suggests that providing ARVs to children in a health center/nurse-based program is both feasible and very effective. Adequate numbers and training of nursing staff and an emphasis on the psychosocial needs of caregivers and children have been key elements for the successful scaling-up of ARVs at this level of the health system.

## Background

Treatment of children with the acquired immunodeficiency syndrome (AIDS) using antiretroviral drugs (ARVs) has been a major challenge in the fight against the human immunodeficiency virus (HIV), especially in resource-constrained settings. In many African countries, the shortage of human resources for health is one of the major barriers to achieve universal access to HIV treatment and care. In particular, reliance on doctor and hospital-centered care hampers the ability to scale-up antiretroviral treatment (ART), and task shifting, the process of delegation of tasks to health workers with lower qualifications, has become a recognized strategy [1-4].

While there have been a number of studies showing good outcomes in treating pediatric populations with ARVs in resource-constrained settings [5-16], these programs have essentially been carried out in hospital settings and/or with significant physician involvement. Although nurse-based ART provision in health centers could increase the coverage of ART, it is not clear whether this compromises quality in terms of outcomes. There are no published studies demonstrating detailed methods and treatment outcomes of children in health centers staffed by nurses. In particular, there is limited published information on how to address the psychosocial issues related to HIV, a major challenge for pediatric ART programs [17], in settings with few specialized personnel available.

As in most African countries, Rwanda has an acute shortage of physicians and is relatively better resourced with nurses [18]. In a recent simulation model, it was estimated that, relying on a physician-centered service provision model, 51% of the total physician capacity of the government of Rwanda would be absorbed by HIV care and treatment by the end of 2008 [19]. Thus, it is completely logical to make greater use of nurses to manage HIV care, but the question remains whether doing so leads to good quality outcomes, especially for pediatric patients.

This report describes the nurse-centered pediatric ARV program implemented in two government health centers in Kigali, Rwanda, with details of its psychosocial aspects and treatment outcomes.

## Methods

### Design

Retrospective analysis of routinely collected outcomes from the ARV program in two health centers in Kigali, combined with interviews with key health center and Médecins Sans Frontières (MSF) staff. MSF reports since program inception were also reviewed.

### Setting

Rwanda, with a population of around 9 million inhabitants, has an overall HIV prevalence of 3% and more than 7% in urban areas [20]. The national ART program, launched in 2003, was first established mostly in the district hospitals, with subsequent decentralization to the health centers. From 2004 on, a gradual scaling-up was seen. The latest estimate in 2006 by TRAC (Treatment and Research AIDS Center) of the number of HIV-infected children was 13,901 with half of them (6951) in need of ARVs [21]. By the end of May 2007, almost half (3255) had benefited from ART.

### Health worker distribution in Rwanda and their roles within the HIV care program

With one physician/50,000 habitants and one nurse/3900 habitants, Rwanda is clearly short of physicians but is relatively better resourced with nurses [22]. In addition, 80–90% of the population is living in rural areas and mainly relies on care from primary health centers, staffed by nurses. Up to now, HIV/ART care delivery has essentially been provided by physicians, with nurses only playing a supporting role (see Table 1). In the traditional model, physicians were responsible for all tasks. Equally, at primary health centers, the bulk of medical care was provided by the visiting physician, who was based in the district hospital. When relating to HIV care for children, this reliance on physicians/hospital-based care was even more pronounced as it was believed to be more difficult care.

**Table 1:** Traditional and modified tasks for nurses and physicians within the HIV/ART care program

	**MD-CENTERED**		**NURSE-CENTERED**	
	**Role MD **	**Role Nurse**	**Role MD **	**Role Nurse**
**Pre-ART care**				
Initial physical exam/staging	X			X
Ordering CD4 count	X	X		X
Assessment of ART eligibility	X			X
FU of non-eligible patients	X			X
CTX refill		X		-^b^
Complex medical cases	X		X	
				
**ART care**				
Ordering lab tests	X	X		X
Interpretation of lab tests	X			X
ART initiation and FU				
Non-complex cases	X			X
Complex cases^a^	X		X	
ART refill		X		-^b^
Register keeping/reporting		X		-^b^
Filing of results/medical records		X		-^b^
Training/mentoring			X	
Supervision			X	

^a ^Complex cases included those with advanced HIV disease, severe/persistent opportunistic infections, severe ART side-effects, suspicion of ART failure, severe or recurrent non-adherence to ART.

^b ^Activities taken over from the ARV nurse by other staff.

ART: antiretroviral therapy; FU: follow-up; CTX: cotrimoxazole; MD: medical doctor

In the Rwandan health system, nurses are classified into three levels according to their level of training: 1) A3 nurses may have no/minimal secondary training and minimal health training; 2) A2 nurses (the bulk of the health workforce) have two years of secondary education and two years of nursing training; 3) A1 nurses have received two additional years of nursing training after finishing secondary school [22]. Whereas initially A1 nurses were rarely found at health centers, this is gradually changing. Currently, no other non-physician clinician cadres are being trained.

### Study sites

The two clinics in this report were Kimironko and Kinyinya health centers, located in Kigali. Kimironko was an urban government health center with a catchment area of about 75,000 people while Kinyinya was semi-rural, being located at the outskirts of Kigali, with an estimated population of 17,000.

In addition to routine health care, the health centers provided comprehensive HIV care and started offering ARVs at a decentralized level, beginning in October 2003 in Kimironko and followed in January 2004 in Kinyinya. They were among the first services in the country to offer ART. By July 2007, 3252 patients had been started on ART within these two clinics and of these, 332 children were enrolled in the ART program. These two clinics have been supported by MSF since 2002.

### Study population

**Figure 1:**
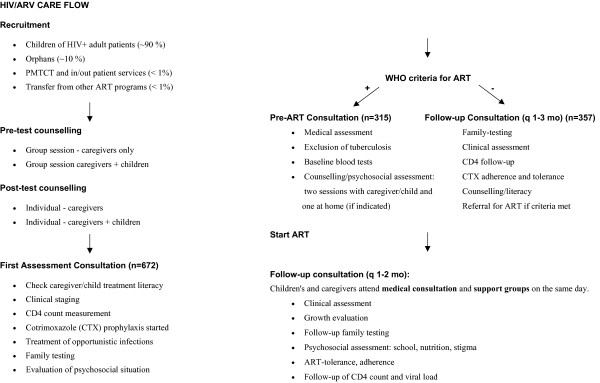
**Flow diagram of the pediatric HIV program**. ART: antiretroviral treatment; PMTCT: Prevention of Mother To Child Transmission; WHO: World Health Organization.

The analysis included all children enrolled in the HIV program who qualified for ARVs from the launch of the ART program from October 2003 till Jan 1/2007. With data collected until June 30/2007, all children had been on ART for at least six months. See Figure 1 for details about how children were selected for ARVs and their treatment paths.

### Description of the Pediatric HIV program

#### National government's commitment and external support

The pediatric program in the two clinics was part of the Rwandan national ART program, essentially run by government health care staff. The national program has seen a successful scaling-up over the last years, organized through the National AIDS Control Commission (NACC) and TRAC. A constructive collaboration with various international partners has taken place, with substantial financial support provided by the Global Fund to fight AIDS, Tuberculosis and Malaria. The government's commitment was demonstrated by providing additional nursing and laboratory staff as well as ongoing laboratory services, training and ARV procurement. MSF's contributions were essentially aimed at increasing the clinics capacity for ARV care and included training and supervising staff, refurbishing clinic buildings, upgrading the labs and providing financial incentives.

#### Nurse-based treatment and care

##### Increase and retention of nursing staff

At the launch of the HIV program in 2002, the health centers were poorly staffed. The increased staffing by the Ministry of Health was a critical factor. Whereas the health center's staff included 7 trained nurses before the launch of the HIV program in 2002, this had gradually increased to 28 by the end of 2004 (mainly A2 nurses), when scaling-up began. Overall, around 50% of the entire staff's time was dedicated to HIV care. To compensate for the substantial increase in work-load associated with the fast scaling-up of the ART program, a performance-based financing mechanism was put in place, initially backed by MSF.

To avoid overloading the nurses, most of the tasks traditionally performed by nurses were taken over by new or reinforced cadres in the health centers: receptionists for administrative work and data collection/monitoring; counsellors and community support groups for counselling; and lab-staff for blood collection.

##### Training for nurse-based treatment and care

When the ART program was launched, each health center was supported by a HIV physician. Given the high number of patients in (urgent) need of ART, one of their main duties was to build local capacity, facilitating a rapid scaling-up of HIV treatment and care within the health facilities. From the onset, nursing staff received theoretical and bed-side training in comprehensive HIV care in general, and in pediatric aspects in particular, from the HIV physician, gradually increasing their knowledge and confidence. Using this knowledge, they were able to initiate and change ARV treatment (for toxicity or contra-indications), with medical confirmation, and perform routine follow-up of the children, aided by simplified treatment protocols and growth curve charts provided in the consultation rooms. Protocols for recognizing side-effects of ARVs were standardized and indications for referral to the physician were defined. Ongoing training and supervision was ensured by having one full-time equivalent physician per health center, who was ultimately responsible for the medical care. As the program matured, physician involvement decreased to a presence two to three times a week. A great deal of emphasis was placed on comprehensive care with a family-centered approach including methods to address the psychosocial issues of HIV. ARVs were dispensed by a nurse with extensive practical experience in ARVs and additional training through the national program.

##### Mentoring and supervision

Besides consulting the complex/referred cases, the physician played a major role in assuring the quality of care provided in the program (Table 1). The physician did not have a separate consultation office, but reviewed patients together with the nurses, allowing for ongoing training and skills-building. In addition, this permitted ongoing evaluation of the quality of care provided by the nurses, and to identify areas where additional training was needed. At the same time, a sample of patient files was reviewed to assess completeness of care and to trace medical problems. Only if the physician was confident in their knowledge and skills, were nurses allowed to consult in the ART program, but they still continued to receive ongoing training and supervision.

#### Voluntary Counseling and HIV testing (VCT) for children: overcoming barriers among caregivers

Early in the program, we observed that barriers existed among the caregivers towards testing their children. Caregivers themselves were distressed by their own recent HIV diagnosis. They were reluctant to discuss testing of their children, since they felt guilty, fearing the reaction of the child to their own and the child's disclosure, and being worried about the health and future of the child [23-25]. Thus, from the start of the program, support and discussion groups were organized for the caregivers, designed to increase their acceptance of their HIV-positive status, a prerequisite for discussing testing of their children. In addition, prior to starting ARVs, individual in-depth counseling sessions were held with the caregivers to discuss testing of children in detail [23,25]. Subsequently, the health center-based support groups were transformed into community-based support groups. Within these, the group leaders, serving as role-models of positive living with HIV, helped to make HIV more easily discussed within the community and to raise issues like testing of partners and children.

#### Timely and careful disclosure and ongoing psychosocial support

**Figure 2:**
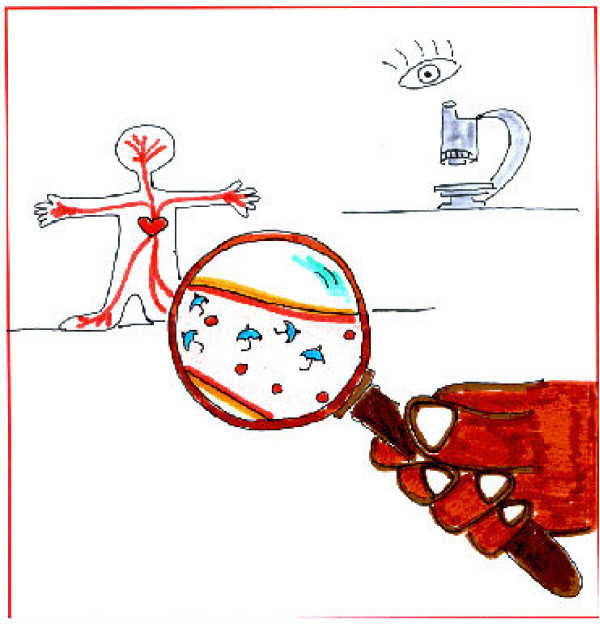
**Picture from the booklet used to explain HIV/AIDS to children**. The booklet is used by caregivers and health center staff. This picture is used to explain "what actually is blood ?" to the child.

In our experience, children were generally not well prepared when they came for testing. Few children knew exactly why they were there, had little knowledge of HIV and rarely knew about the status of the caregiver [25,26]. However, the children preferred to be informed about their and their caretaker's status from the caretaker and felt cheated when they were not told the truth. Consequently, we tried to involve the caretaker as much as possible during disclosure. Caretakers were first counseled on why it was important to talk openly with their children about HIV, why their active participation was important for the child, how the child might react, and how they should respond to questions. During disclosure, we tried to have the caregiver explain their and the child's status, while being supported by the counselor [26,27]. The use of a booklet explaining HIV provided a common language around HIV (Figure 2).

Testing of children was done on a dedicated day, ensuring that enough time was available for every child, making the environment/event more child-friendly and facilitating on-site training of health care staff in counseling of children. Disclosure was considered when the child was > 6 years old [27,28].

Within the child support groups, an environment was created by the counselors where the children could express themselves, raise their questions and worries and develop a positive attitude towards life with HIV. Most of the issues discussed were raised by the children themselves and reflected their deeper feelings: HIV (what, why and how?), life and death, sexuality, manipulation in the caregiver-child relationship and discrimination. The issues were addressed in several ways using open discussions, games, fairy-tales and drawings [27,28]. These groups (consisting of 12–15 children) were organized according to age and were open to all children from the age of 7 years on, irrespective of taking ARVs. Our approach was based on general recommendations available within MSF [27] that were adapted to the local context and taken into account the capacity/possibilities within the program.

Initially, children were followed medically together with the adult patients, but had their support groups on separate days. Subsequently, a special consultation for children, integrating both medical and psychosocial aspects, was introduced once a week, reducing the amount of traveling and out-of-school time for the children. It also allowed them to receive care and follow-up in the presence of other children, making the whole experience more child-friendly [27].

#### Who were the caregivers ?

Close to 65 % of the children were taken care of by one or both parent(s). Twenty-five percent received care from the extended family (grand-parents, brothers/sisters, cousins,...), and about 10 % of children were orphans with adoptive caregivers. In general, no child was tested without the caretaker being tested.

### ART eligibility, regimens and safety

Children were started on ART according to the national guidelines, based on the World Health Organization (WHO) immunological and clinical eligibility criteria and using WHO-recommended first line regimens [29]. To simplify treatment regimens and limit calculation errors, four weight categories were used to choose the appropriate dosing of the ARVs, as has been described elsewhere [9,30]. Generic, scored fixed-dose combinations (FDC) were prescribed as much as possible (Triviro 30/40, Ranbaxy), containing stavudine/lamivudine/nevirapine. No quarter-tablets were used (as use of these had not been validated), only whole or half tablets were prescribed. When scored FDCs were not available, children were given either separate medications or were switched to a zidovudine-containing FDC (Duovir-N, Cipla). Initially, small children needing syrup formulation were referred to the central referral hospital in Kigali (CHUK) but from mid-2005, the syrup was available at the health center level.

For most children (orphans in particular), a home-visit was done before ARV initiation to assess the socio-economic situation of the family and to provide support where necessary. Adherence to treatment was evaluated by self-report using calendars, regular clinical attendance (monthly pharmacy refills/visits after start-up period) and pill counts. As well, viral load counts were performed routinely after the first year of treatment from 2005 [31]. HIV care, including ARVs, consultations, lab tests and OI medications, were provided free of charge. Transport costs for the children were covered for families in need.

Side effects were evaluated at each follow-up visit, and treatment changes were made for grade 3 and 4 events [29]. Liver function tests were performed at baseline, 2, 4, 8 weeks and then every 6 months and on clinical grounds. A full blood count was done at baseline, 12 weeks and then every 6 months and on clinical grounds. For children on zidovudine, an additional control was done at 4 and 8 weeks.

At the outset, absolute CD4 counts and percentages, checked every 6 months, were measured with the FACSCalibur™ Flow Cytometry System (Becton-Dickenson) at the National Reference Laboratory (NRL). From 2003–2004, the FACSCount apparatus (Becton-Dickenson) was used and percentage counts were calculated based on total lymphocyte counts of a separate sample.

CD4 percentage counts at baseline were missing for several reasons (n = 33). First, due to limitations in the number of samples that could be handled at the NRL in the initial stages of the program, some children had no baseline CD4 counts because they met clinical criteria for starting ART. Second, for some children transferred from other sites, the information was unavailable. Finally, if the total lymphocyte count was not known, the CD4 percentage could not be calculated, and absolute CD4 counts were used for consideration of ART. The median time from baseline CD4 sample collection to initiation of ARVs was 53 days (interquartile range 30–79). Viral loads were analyzed at the NLR by the Ultrasensitive Amplicor assay, version 1.5 (Roche). All other biochemical tests were performed at Kinyinya health center.

### Data collection and statistical analysis

Data were routinely collected during the consultations using Access^® ^software. Treatment and safety outcomes of the cohort were analyzed by Stata software 9.0^® ^(STATA Corp., College Station, Texas, USA). Kaplan-Meier survival methods were used to estimate the probability of remaining in care (failure defined as death or lost to follow-up). CD4 percentages were used for children under 5 years to evaluate immunological status and both CD4 counts and absolute counts used for children above 5 years. Weight-for age Z-scores (WAZ) were calculated with Epi-Info (version 4.3.1; Centers for Disease Control and Prevention). Data for this study were collected from October 1/2003 until June 30/2007.

### Ethics

This study analyzed routinely collected data from the pediatric HIV program. Approval for publication of this data and report was received from the NACC and TRAC. Confidentiality was maintained with no patient identifiers being used. The Ethics Review Board of MSF and the Rwandan National Ethics Committee gave exemption from formal ethical review. No informed consent was obtained for this study.

## Results

### Characteristics of the study population

Over a period of about 3 years, 315 children were started on ARVs, with a median age of 7.2 years. Table 2 describes their characteristics. Approximately 25% were less than 5 years of age, 7 were started before the age of 18 months. Sixty percent were in WHO stages I and II at treatment initiation. Fifteen tuberculosis (TB) cases were recorded of which 4 had extra-pulmonary manifestations. Baseline CD4 counts percentages were available for 282 children with a median value of 14%. The median WAZ-score was – 1.9. The vast majority of children, 89%, started on stavudine/lamivudine/nevirapine, with adult FDC tablets used for 282 children. The remaining 33 commenced syrup formulation of whom, by the time of analysis, all but five had changed to tablets. The median time on ART was 2 years. Four fifths of all children were followed for more than one year with a total of 589 patient years of follow-up.

**Table 2:** Characteristics of children started on antiretroviral treatment (n = 315)

Age at start^a^	7.2 (4.5 – 10.4)
< 3 years^b^	38 (12%)
3 – 4.9 years^b^	51 (16%)
5 – 14.9 years^b^	226 (72%)
Sex (male/female)^b^	157/158 (50/50%)
Clinical WHO-stageq^b^	
WHO stage I	43 (13.7%)
WHO stage II	145 (46.0%)
WHO stage III	115 (36.5%)
WHO stage IV	12 (3.8%)
Weight for age (Z-score) (n = 293)^a^	-1.9 (-3.0;-0.9)
Baseline CD4 count % (n = 282)^a^	14 (9–18)
< 15%^b^	158 (56.0%)
15–25%^b^	118 (41.8%)
> 25%^b^	6 (2.1%)
Baseline absolute CD4 counts (n= 302)^a^	345 (229–572)
Baseline hemoglobin (mg/dl)(n = 268)^a^	11.0 (10.3–11.8)
ART regimen^b^	
d4T/3TC/NVP	281 (89.2%)
d4T/3TC/EFV	6 (1.9%)
AZT/3TC/NVP	19 (6.0%)
AZT/3TC/EFV	9 (2.9%)
Time on ART (years)^c^	2.0 (1.2–2.6)
Total patient years of follow-up	598
On tablets (FDC)	577
On syrup	21
< 1 year vs. ≥ 1 year^b^	59 (19 %) vs 256 (81 %)

^a ^Values are expressed as median (interquartile range)

^b ^Values are expressed as n (%)

^c ^As of June 30/2007 (closing date of dataset)

WHO: world health organization; ART: antiretroviral treatment; d4T: stavudine; 3TC: lamivudine; NVP: nevirapine; EFV: efavirenz; AZT: zidovudine; FDC: fixed-dose combination

### Clinical, immunological and virological outcomes

**Figure 3:**
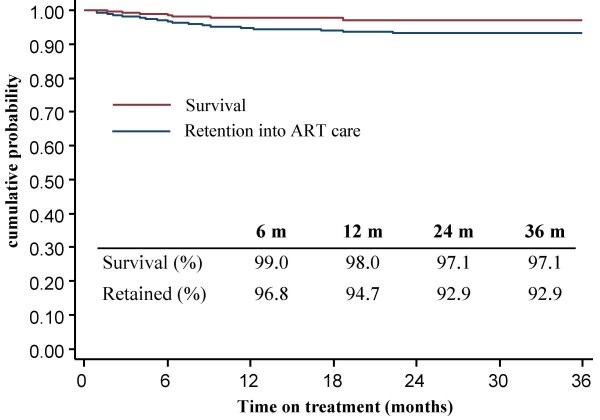
**Kaplan Meier curve depicting the probability of survival or retention into the antiretroviral treatment (ART) program (n = 315)**. Children that had died or were lost to follow-up were considered as non-retained into ART care.

By June 2007, 84% (265) were still alive and being followed-up in the clinics. Thirty (9.5%) had been transferred to another health facility offering ART. Eight (2.6%) had died, only 12 (3.8%) were lost to follow-up (defined as not coming to their last scheduled visit for more than 2 months). Of the 8 deaths, 2 were clearly not linked to HIV. Kaplan-Meier estimates (Figure 3) showed a probability of remaining in care of 94.7% and 92.9% at 12 and 24 months respectively (with failure defined as death or lost-to follow-up). CD4 results, hemoglobin and WAZ all showed progressive improvement over the treatment interval (Table 3). Most viral load results were obtained between and 15 and 23 months and were available for 87% by 18 months on treatment (n = 174). Viral load was less than < 400 copies/ml in 82.8% of children and showed satisfactory viral suppression in 86.8%. For 13 children among whom viral loads were detectable, a second sample was made available after adherence counseling. The median time from first viral load collection to the repeat sample was 5.8 months (interquartile range 2.7–8.8). Four out of the 13 positive viral loads became undetectable and 1 decreased significantly. Two children were switched to second line treatment. For the remainder, adherence problems were still being addressed or criteria to switch were not met.

**Table 3:** Clinical, immunological and virological outcomes for children on antiretroviral treatment (n = 315)

	**Baseline**	**6 months**	**12 months**	**24 months**	**36 months**
CD4 count					
< 5 years (%)^a^	16	30	32	33	35
IQR	(12–19)	(23–35)	(26–34)	(28–36)	(28–40)
N	84	59	48	39	7
≥ 5 years (%)^a^	13	25	26	29	29
IQR	(9–17)	(18–29)	(21–32)	(24–34)	(19–33)
N	198	175	117	80	24
≥ 5 years (abs)^a^	297	550	624	704	616
IQR	(181–405)	(337–747)	(459–840)	(562–866)	(492–865)
N	217	183	120	80	24
Hb (mg/dl)^b^	11.0	12.1	12.6	12.9	13.7
WAZ^a^	-1.9	-1.6	-1.6	-1.5	-1.5
IQR	(-3.0;-0.9)	(-2.6;-0.8)	(-2.6;-0.7)	(-2.5;-0.6)	(-2.7;-0.6)

Viral load	Months on ART^a^		< 40 c/ml^c^	< 400 c/ml^c^	< 5000 c/ml^c^
(n = 174)	18 (15–23)		127 (73.0%)	144 (82.8%)	151 (86.8%)

^a ^Values are expressed as median (interquartile Range (IQR));

^b ^Values are expressed as median;

^c ^Values are expressed as n (%)

abs: absolute CD4 count (in cells/μL); Hb: hemoglobine; WAZ: Weight-for-age Z-score; ART: antiretroviral treatment

We only had complete data on pharmacy refill as an indirect measure of adherence to therapy. Allowing a delay of up to two days (accounting for the security stock), we defined excellent, good and poor adherence as being punctual for > 95%, 80–95% or < 80% of the visits respectively. Poor adherence was observed for 5% of the children, with the majority having excellent (49%) or good (46%) adherence.

### Safety and tolerance

Among all children placed on ART, a change in therapeutic regimen was required for 46 children (14.6%), out of whom only 26 (8.3%) were due to toxicity. The start of TB treatment provoked 18 switches. Toxicity was mainly related to nevirapine, requiring a treatment change to efavirenz (n = 24). Sixteen cases were due to grade 3–4 skin manifestations of which two were Stevens-Johnson syndromes occurring within 1 month of therapy. Both recovered. Five early changes (within 3 months) were made for severe hepatitis, with 4 children showing clinical signs, and 1 child having asymptomatic grade 3 liver toxicity. An additional three children were changed late (median 9 months) due to symptomatic hepatitis. We observed an additional 14 children with grade 2 (n = 11) or grade 3 (n = 3) liver toxicities, mostly occurring within the first months of ART. In general, these were transient and well tolerated and did not require treatment change.

Stavudine was changed for one child with severe neuropathy, and two cases of lipoatrophy were reported (one requiring treatment change). No lactic acidosis or anemia requiring treatment change were observed. Thirteen children were found with a hemoglobin level < 7 mg/dl of which 5 were present at baseline and generally improved on stavudine-containing ART. Of the others, none were related to zidovudine and were transient and/or related to intercurrent infections.

## Discussion

This report offers reassuring evidence that health center/nurse-based ART delivery for children is both feasible and very effective. The morbidity and mortality results are comparable to or better than those of other reported studies, which are essentially hospital-based [5-16]. One of the strengths of the study is that the therapeutic responses were confirmed with virological evidence in the majority of children. This is very important since it suggests that clinic-based programs can provide high quality care without over-reliance on the most sparse human resources in the health system and is thus more likely adapted to and sustainable within this context. In addition, decentralized/nurse-based care might have potential advantages over hospital/physician-centered care in providing accessible and comprehensive HIV care to children.

### Reinforcing nurse-based care

The shortage of physicians in many African countries requires a more strategic use of their skills, without compromising quality of care. The health center-based care described here was provided to a large extent by nurses, with appropriate physician supervision. A most important factor in the success of the program was the increase in number of nurses, a human resource pool relatively more available in Rwanda and many African countries, in the health facilities by the Ministry of Health. A substantial part of the physician's role was to ensure ongoing on-site training, mentoring and supervision, to gradually increase the capacity and skills of the nurses. Although considerable energy and time were spent on this, especially early in the program, in our experience it clearly paid off in the end. Capitalizing on the potential of the nurses allowed for a rapid scaling-up that would have been difficult, if not impossible, to achieve when relying on physicians only for ART care. At the same time, we would like to caution against over-reliance on nursing skills for ART care in the absence of adequate supervision [32]. We believe that the complementary interplay between nurses' and physicians' skills has been important to achieve good results.

Another important element of success has been the high retention of nurses. We think that the main contributing factors have been the continuous training and skills building, the high quality of care provided and patients' satisfaction, the increase in nursing staff to meet the demand, the promotion of responsibilities for the staff in the project and the performance-based financial incentives [4,33,34]. In addition, the creation of new support staff that took over some nursing duties has avoided overloading the nurses. This has allowed a steady increase in the pool of nurses skilled in HIV care. Over time, consulting nurses with 3–5 years experience in ART care have become more involved in the training of other nurses, and currently function as a source of reference. Their experience has also allowed a gradual decrease of physician involvement to a presence two to three times a week.

### Decentralized care: increased accessibility and acceptability

Centralized HIV care at the hospital level tends to create additional bottlenecks in treatment initiation, and creates difficulties with transport and time off work for the patients [29,35,36]. It has been observed in other settings that, relative to nurse/health center based ART delivery, the overall capacity of hospital/physician based ART care in number of patients that can be treated is far more limited, which obviously raises concerns about access to and quality of care provided [34]. The fact that this program was based at a decentralized level might have contributed to earlier diagnosis and good adherence in the program in a number of ways. First, although the distance to the closest hospital was not enormous given the urban setting (1–2 hours walk), geographical proximity is still likely to have facilitated increased access and acceptability for both caregivers and children, and this is consistent with findings from other studies [37]. This will obviously be more important in rural settings. In addition, we were able to provide comprehensive and patient-centered care, within a family-based approach, closely linked with the community. Having all staff trained in HIV/ARV care meant that they were sensitized to the issues in all service areas. Care and counseling could be provided at every patient encounter, and were not limited to the physician's consultation or a specific service as is often the case in the hospital setting. Convenient scheduling of visits also facilitated accessibility as did comprehensive care that was provided free of charge [37]. The very low rate of lost to follow-up observed in our program seems to confirm that services were 'user friendly'.

### Nurse-based care – multiplying care capability

By 2005, physicians constituted 4.2% of the total health work force in Rwanda, 52.2% were nurses [22]. Recent estimates from Rwanda suggest that approximately one full-time equivalent physician would be needed for 500 patients on ART within the traditional ART care model [19]. A simulation model for ART care delivery in Rwanda estimated that allowing nurses to initiate and follow the ART treatment for non-complex cases would see a 76% reduction in physician demand for HIV care or an 170% increase in physician capacity for non-HIV care [19]. Within the nurse-centered model, as described here, we equally observed a gradual decrease in the need of physician time to approximately one full-time equivalent physician for 1500 patients after the first capacity had been built within the health centers and one equivalent for 3000 patients at later stages. Although investment in human resources at all levels remains a priority, nurse-centered ART care constitutes a more rational use of the human resources currently available.

### Psychosocial Care

We started ART in a relatively healthy cohort of children, as seen from the clinical and immunological baseline data. This, in turn, was due mostly to our identifying children earlier in their disease. From the outset, a family-centered approach was implemented with a strong focus on the psychosocial aspects of HIV care (Table 4). This played a crucial role in overcoming barriers to routine testing of children. Dealing with the HIV-infected adults' psychosocial issues allowed the staff to introduce the idea of testing children, and helped them prepare the children for disclosure [25,38]. Although it remains hypothetical, we think that this sensitive approach, combined with the goal of routinely testing all children of adult HIV-infected patients, facilitated the early diagnoses reflected here. Our experience also demonstrated that there was a significant burden of HIV illness in children that was readily identifiable through HIV-infected adult caregivers. Over 90% of the children in this cohort came from this source.

**Table 4:** Psychosocial aspects of the pediatric HIV program

**Caregiver-centered approaches**
- organization of discussion/support groups, at health center and in community
- family-based approach to identify eligible children
- individual counseling (pre-ART)
- psychosocial issues addressed in follow-up care
**Child-centered approaches**
- adapted counseling for children for disclosure and ART (child-adapted tool)
- designated days for children's clinics
- child support groups
- integrated care, including disclosure, with their caregivers
**Health-staff centered approaches**
- discussion groups for health care staff
- training on psychosocial implications of HIV
- practical training by psychosocial team (check-lists,...)
- supervision and mentoring

ART: antiretroviral treatment

HIV: human immunodeficiency virus

In addition, once children tested positive, the psychosocial emphasis provided effective means for handling disclosure and subsequent commencement of ARV treatment. Preparation of both caregivers and children contributed to good adherence and treatment outcomes [24,39]. This supports previous research that emphasizes that HIV care is more than getting CD4 counts and prescribing ARVs. Addressing the many psychosocial aspects of the illness is crucial to effective treatment programs [23,38,40]. Whereas this has been effectively implemented in high-income countries and in some hospital-based settings in low-income countries, we think this aspect of pediatric ART has not received sufficient attention in decentralized contexts [17]. Our program experience emphasizes the need and feasibility of providing psychosocial support within health center-based pediatric ART programs.

### Safety

In our program, ART was safe and very well tolerated, with few severe side-effects reported. In concordance with others [5,12], tolerance of ART seemed to be as good or better in children than in adults, arguing against the need for specialist care for pediatric ART and in favor of providing ART treatment to children at a decentralized level. In addition, treating a healthier population was also likely to reduce side-effects.

Virtually all treatment changes for side-effects or contra-indications were initiated by the nurses, and confirmed by the physicians, in patients with clinically recognizable symptoms. This is reassuring for decentralized pediatric treatment in rural areas where laboratory monitoring might not be available. Few children had to change therapy due to rashes or hepatitis. No adverse effects resulted in death. No cases of severe anemia or lactic acidosis reported, while neuropathy and lipoatrophy were extremely rare. One could argue that the low rate of side-effects could be explained by their having been missed by the nurses. However, within this program, side-effects were documented and treated by the same nurses who managed our adult cohort [41,42], at a frequency similar to other studies [35,43-45]. Although diagnosing ART toxicity in children might be more challenging, children were additionally monitored through chart review and virtually all were seen by a physician at some point in time. Side-effects missed by the nurses were extremely rare.

### Replication/sustainability

We are confident that this kind of program could be replicated in other settings and is sustainable. The program was run in health centers that were already providing a full range of primary care services. The success of the program depended, to a large extent, on the commitment of the government. The increased staffing was a critical factor. External support provided by MSF was essentially directed at capacity building (training, infrastructure, logistics) and has gradually been taken over through the maturing national ART program. The financial incentives provided by MSF were subsequently replaced by a performance-based pay initiative of the Ministry of Health, gradually implemented since 2005 [46]. In addition, all nurses in the public sector have seen a substantial increase in salary. The supervising and mentoring role of the health center-based physician has been gradually taken over by the visiting district physician. This transition has been aided by additional training and quality assurance provided by the national ART program and performance-based financing. The external support decreased gradually over the years, and MSF handed over the project to the Ministry at the end of 2007. Although a follow-up visit by mid 2008 could not reveal any major problems subsequent to the hand-over, this should ideally be reassessed in a few years time.

Another program in Rwanda recently reported excellent patient outcomes for their adult population and good compliance with national guidelines using a health center/nurse-based model [47]. The national program is currently considering expanding this model of care at the national level.

It is important to note that this program, especially the psychosocial elements, was developed over several years and in response to perceived needs of the children and caregivers. This helps explain the ongoing changes we describe, reflecting the subsequent barriers we encountered regarding testing and treatment of children. It is not necessary that the full structure described here be operational at the outset of a program, but could be developed over time, depending on facilities and staffing availability and on the evolving challenges.

### Remaining challenges

While the program successfully managed to build nursing skills and to redefine the role of the physician, high quality of care will require further training for nurses and redefinition of the tasks and responsibilities of the nurses and physicians, based on the evolving challenges. Treatment failure, an inevitable clinical challenge for the future, in particular, will require a careful mix of physicians' and nurses' skills. In addition, increasing problems of acceptance and adherence can be expected in the long run, especially during adolescence. To what extent these will be successfully managed within the program remains to be seen.

Given the severe lack of human resources for health care at all the levels in most low-income countries and the pronounced increase in need to achieve universal access to HIV care, task shifting can only be successful in the long-term when combined with investment in human resources at all levels [32].

### Limitations

Given our relatively healthy cohort, we are cautious in suggesting that similar outcomes would be possible with a sicker population. Since CD4 counts were not available for all children, and some children were started on clinical grounds, the missing baseline CD4 counts might have biased the baseline values to some extent. Consequently, it is possible that the comparison between baseline and follow-up CD4 counts is not entirely accurate, but we believe the differences would be minor. Similarly, we only recorded 87% of viral loads by 18 months. Given the similar baseline characteristics, we have no reason to believe that the results of patients with missing values would be substantially different. We also note that this cohort included few young children and we cannot be sure if similar outcomes would apply to them. At the early stages of the program, the youngest children depended on the university hospital to receive their ARVs in syrup formulation before it become available at the health center level. Since this only involved 9 children, we do not think this could significantly affect the program outcomes. The health centers received targeted support from MSF that has been gradually withdrawn. Although the program seems to be sustainable, with quality of care remaining high, its future success will depend on the governments' ongoing commitment and resources.

To what extent local factors influenced the outcomes of the program remains to be determined. This program was part of and has benefited from the successful scaling-up of the Rwandan National HIV program. The HIV prevalence rate in Rwanda is lower than most Sub-Saharan African countries. The context of HIV has certain particularities linked to the history of genocide.

Finally, although the program appeared to have been successful, at least in treatment outcome terms, caregivers' and children's perceptions and satisfaction were not known and should be assessed. This information could potentially be an important determinant of long-term success of pediatric ART programs, where the psychosocial consequences of HIV and the overall impact on childrens' lives might be even more pronounced than for adults [17].

### Future research

This study points to further research to see whether this health center model could be successful with sicker and/or younger populations. Whether this approach of decentralized nurse-based pediatric ART programs can be replicated in other contexts remains to be established. Qualitative research into understanding how the psychosocial aspects of care were received would be useful to refine this part of the program.

## Conclusion

Our program shows that, given a strong psychosocial emphasis, health center/nurse-based ART delivery for children is both feasible and very effective, and can at the same time avoid barriers to care related to the scarcity of physicians. Providing comprehensive care close to the community can play an important role in achieving universal access to HIV care for all children.

## Competing interests

The authors declare that they have no competing interests.

## Authors' contributions

All authors read and approved the final manuscript. JVG and LDN were physicians treating the children, CG was coordinator of the MSF HIV project. JU worked as psychologist within the pediatric program. JVG, LDN, JU and CG conceived the project. JVG performed the data analysis and the program evaluation/description; JVG and TR co-drafted the manuscript. LDN, AA, CG and JU critically reviewed the manuscript and improved the intellectual content; TR performed the editing.

## Pre-publication history

The pre-publication history for this paper can be accessed here:


